# Cardiac Glycoside 3β-Bufalin Suppresses Cancer Cell Proliferation by Coupling with Na^+^,K^+^-ATPase and Volume-Regulated Anion Channel Within Membrane Microdomains

**DOI:** 10.3390/ijms27177969

**Published:** 2026-09-07

**Authors:** Takuto Fujii, Takahiro Shimizu, Yasuharu Shimizu, Mizuki Katoh, Tomoyuki Okumura, Tsutomu Fujii, Hideki Sakai

**Affiliations:** 1 Department of Pharmaceutical Physiology, Faculty of Pharmaceutical Sciences, University of Toyama, Toyama 930-0194, Japan; takshimi@pha.u-toyama.ac.jp (T.S.); kato3mizu@shinshu-u.ac.jp (M.K.); 2 Tokyo Research Center, Kyushin Pharmaceutical Co., Ltd., Tokyo 166-0012, Japan; y-shimizu@kyushin.co.jp; 3 Department of Surgery and Science, Faculty of Medicine, Academic Assembly, University of Toyama, Toyama 930-0194, Japan; okumura@ctg.u-toyama.ac.jp (T.O.); fjt@med.u-toyama.ac.jp (T.F.)

**Keywords:** bufadienolide, bufalin, cancer, Na^+^,K^+^-ATPase, membrane microdomain, volume-regulated anion channel

## Abstract

Bufadienolides are toad-derived cardiotonic steroids with anti-cancer activity; however, their Na^+^,K^+^-ATPase-mediated anti-cancer mechanisms remain incompletely understood. Here, we compared the anti-proliferative effects of six bufadienolides, bufalin (3β-bufalin), resibufogenin, cinobufagin, telocinobufagin, cinobufotalin, and desacetylcinobufagin, on human colorectal cancer HT-29 cells. Among them, only 3β-bufalin significantly suppressed cell proliferation in a concentration-dependent manner, with an IC_50_ of 1.8 nM, whereas its hepatic metabolite, 3α-bufalin, showed markedly weaker activity, with an IC_50_ of 488 nM. 3β-Bufalin inhibited Na^+^,K^+^-ATPase activity with an IC_50_ of approximately 37 nM, indicating that it suppressed growth at concentrations lower than those required for pump inhibition. The Na^+^,K^+^-ATPase α1-isoform can function as a receptor-type (non-pumping) signaling platform linked to volume-regulated anion channel (VRAC) activation in membrane microdomains of cancer cells. 3β-Bufalin activated the VRAC with an EC_50_ of 4.6 nM, whereas 3α-bufalin and the other bufadienolides showed no detectable activation. The pharmacological inhibition of the VRAC and the disruption of cholesterol-rich membrane microdomains significantly attenuated the anti-proliferative effect of 3β-bufalin. Furthermore, 3β-bufalin induced G2/M cell cycle arrest, which was attenuated by VRAC inhibition. These findings indicate that 3β-bufalin selectively suppresses cancer cell proliferation by coupling with receptor-type Na^+^,K^+^-ATPase to activate the VRAC in membrane microdomains rather than inhibiting Na^+^,K^+^-ATPase pump activity.

## 1. Introduction

Cardiotonic steroids are naturally occurring compounds that interact with Na^+^,K^+^-ATPase and have long been recognized for their effects on cardiac contractility. They are broadly classified into two structural groups: cardenolides and bufadienolides. Cardenolides, such as ouabain, digoxin, and digitoxin, possess a five-membered unsaturated lactone ring at C17 of the steroid nucleus and are found mainly in plants. In contrast, bufadienolides, such as bufalin, cinobufagin, and resibufogenin, possess a six-membered doubly unsaturated lactone ring and are characteristic constituents of toad venom and related traditional medicines, including Chansu and Huachansu [1,2,3]. Thus, cardenolides and bufadienolides share the ability to bind Na^+^,K^+^-ATPase but differ in lactone ring structure, natural origin, and chemical diversity.

In addition to their cardiotonic effects, cardenolides and bufadienolides have been investigated as potential anti-cancer compounds. Cardenolides have been reported to suppress the proliferation and metastasis of cancer cells and to induce apoptosis and immunogenic cell death through Na^+^,K^+^-ATPase-related mechanisms [4,5,6,7,8]. Bufadienolides have also been reported to exert anti-cancer effects in various cancer types, including proliferation suppression, apoptosis induction, and migration and invasion inhibition [9,10,11,12,13]. Although these findings support bufadienolides as potential anti-cancer compounds, the mechanisms by which they act on Na^+^,K^+^-ATPase remain incompletely understood. 

The Na^+^,K^+^-ATPase α1-isoform is not only an ion pump that maintains Na^+^ and K^+^ gradients across the plasma membrane but also a receptor-like signaling platform organized in membrane microdomains [14,15,16,17]. We previously reported that cardenolide-type cardiac glycosides, including digoxin, digitoxin, and ouabain, activate the volume-regulated anion channel (VRAC) through functional crosstalk with the Na^+^,K^+^-ATPase α1-isoform in the membrane microdomains of human cancer cells [18]. In this mechanism, submicromolar concentrations of cardenolides suppressed cancer cell proliferation without inhibiting Na^+^,K^+^-ATPase activity, which indicates that the receptor-type (non-pumping) Na^+^,K^+^-ATPase contributes to their anti-cancer action. However, whether bufadienolides activate a similar Na^+^,K^+^-ATPase α1-isoform/VRAC pathway remains unclear.

The structural diversity of toad-derived bufadienolides provides an opportunity to examine the influence of chemical features on Na^+^,K^+^-ATPase-mediated anti-cancer signaling. Major bufadienolides, including bufalin, resibufogenin, cinobufagin, telocinobufagin, cinobufotalin, and desacetylcinobufagin, differ in their hydroxylation, epoxidation, and acetylation patterns. In addition, bufalin exists as 3β-bufalin, whereas hepatic metabolism generates 3α-bufalin by inverting the configuration of the C3 hydroxyl group [19,20]. These structural differences may affect their interaction with Na^+^,K^+^-ATPase, ability to inhibit pumping activity, and capacity to trigger receptor-type signaling.

In this study, we investigated the effects of major toad-derived bufadienolides on the proliferation of human colorectal cancer HT-29 cells, with a particular focus on the stereochemical differences between 3β-bufalin and 3α-bufalin. We compared their anti-proliferative effects with their effects on Na^+^,K^+^-ATPase activity and VRAC currents. Our findings indicate that 3β-bufalin is uniquely potent among the tested bufadienolides and that its anti-proliferative action is closely associated with receptor-type Na^+^,K^+^-ATPase-mediated VRAC activation rather than the inhibition of Na^+^,K^+^-ATPase pumping activity.

## 2. Results

### 2.1. Effects of Toad-Derived Bufadienolides on Proliferation of HT-29 Cells

**Figure 1:**
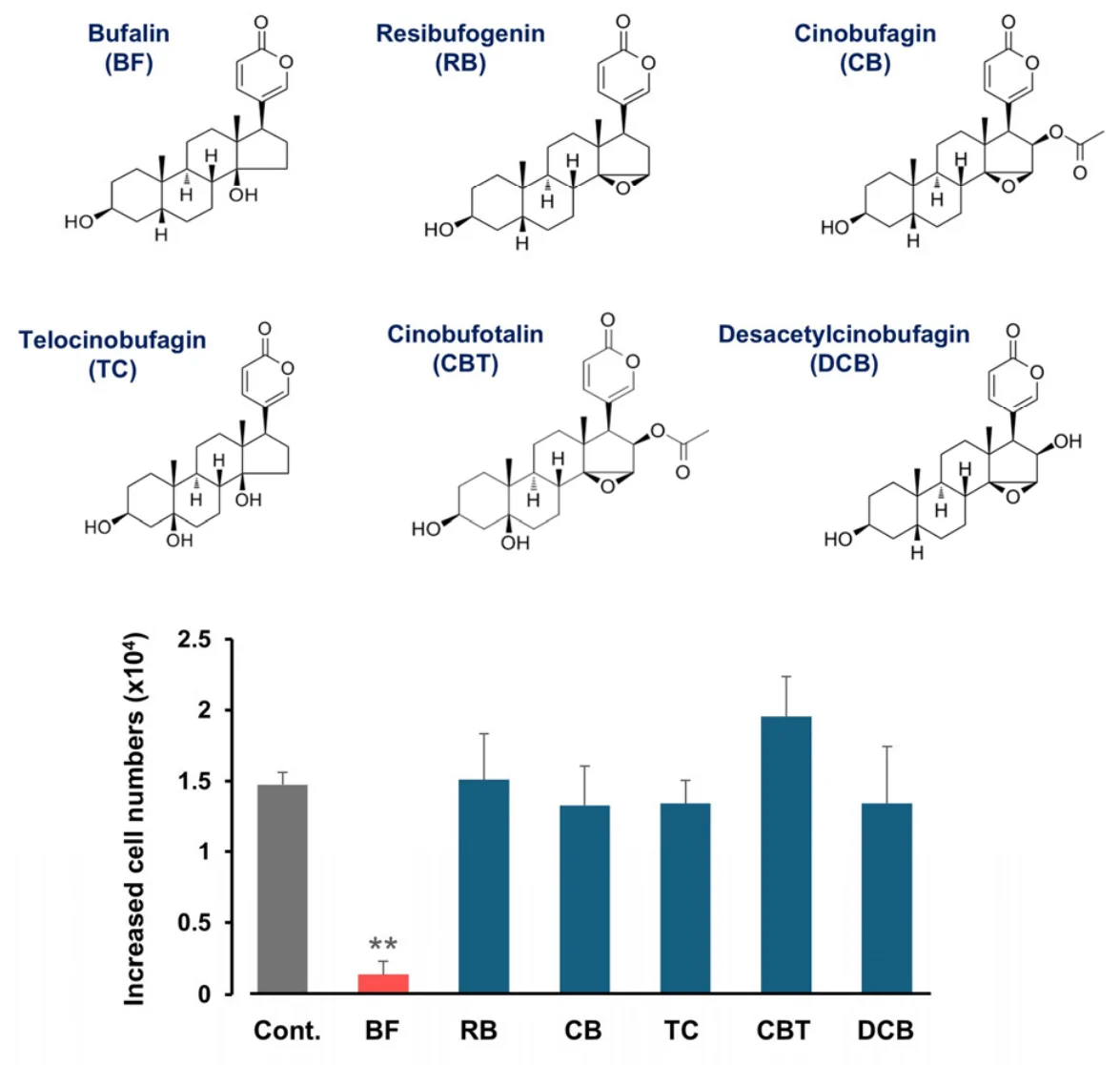
Effects of toad-derived bufadienolides on cell proliferation in HT-29 cells. Cells were treated with bufalin (3β-bufalin), resibufogenin, cinobufagin, telocinobufagin, cinobufotalin, or desacetylcinobufagin at 30 nM for 24 h. Cell proliferation was evaluated by quantifying the increase in cell number. Data are presented as means ± SEM; *n* = 4. ** *p* < 0.01 versus control (cont.).

We first compared the effects of six major toad-derived bufadienolides, i.e., bufalin (3β-bufalin), resibufogenin, cinobufagin, telocinobufagin, cinobufotalin, and desacetylcinobufagin, on HT-29 cell proliferation. Cells were treated with each compound at 30 nM for 24 h, and the increase in cell number was quantified. Among the tested compounds, only 3β-bufalin significantly suppressed the increase in cell number (Figure 1). In contrast, resibufogenin, cinobufagin, telocinobufagin, cinobufotalin, and desacetylcinobufagin exhibited no significant anti-proliferative effects. These results indicate that among the toad-derived bufadienolides tested under these experimental conditions, 3β-bufalin has a uniquely potent anti-proliferative effect.

### 2.2. 3β-Bufalin Is More Potent than Its Metabolite 3α-Bufalin in Suppressing Cancer Cell Proliferation

**Figure 2:**
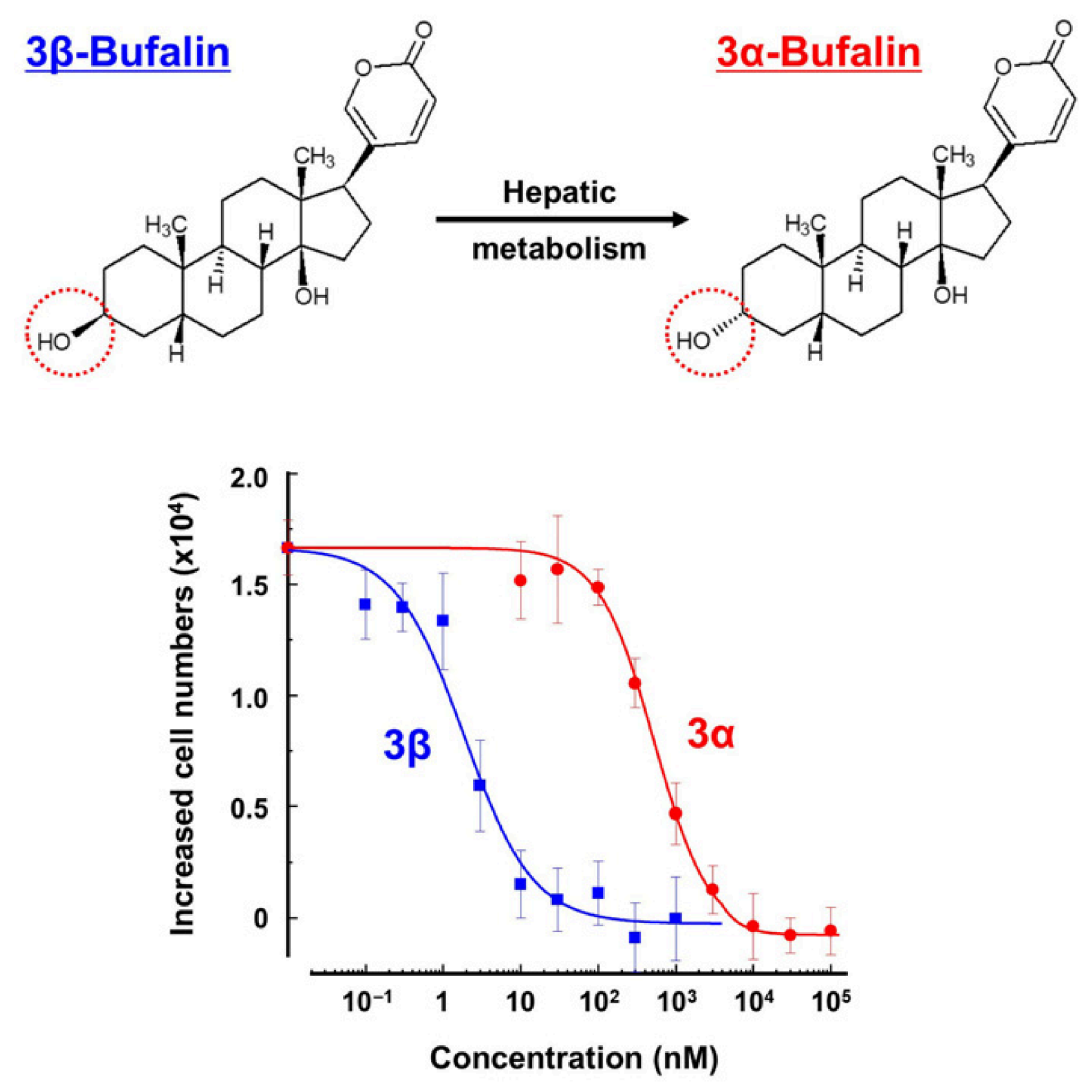
Concentration-dependent anti-proliferative effects of 3β-bufalin and its hepatic metabolite 3α-bufalin on HT-29 cells. Cells were treated with increasing concentrations of each compound for 24 h. Data are presented as means ± SEM; *n* = 4–7.

Because 3β-bufalin alone significantly suppressed HT-29 cell proliferation among the tested bufadienolides, we next examined its concentration-dependent effect in comparison with 3α-bufalin, a hepatic metabolite that differs in the stereochemistry of the C3 hydroxyl group. 3β-Bufalin suppressed the increase in HT-29 cell number in a concentration-dependent manner, with an IC_50_ of 1.8 nM (Figure 2). In contrast, 3α-bufalin showed markedly weaker anti-proliferative activity, with an IC_50_ of 488 nM. 

To examine whether this stereochemistry-dependent difference was limited to HT-29 cells, we also analyzed the effects of 3β-bufalin and 3α-bufalin on human oral cancer KB cells. Similar to the results obtained in HT-29 cells, 3β-bufalin suppressed KB cell proliferation in a concentration-dependent manner, with an IC_50_ of 2.3 nM, whereas 3α-bufalin showed much weaker anti-proliferative activity, with an IC_50_ of 430 nM (Supplementary Figure S1). These results indicate that the β-orientation of the C3 hydroxyl group is important for the potent anti-proliferative activity of bufalin in both HT-29 and KB cells.

### 2.3. 3β-Bufalin Inhibits Na^+^,K^+^-ATPase Activity More Potently than 3α-Bufalin

**Figure 3:**
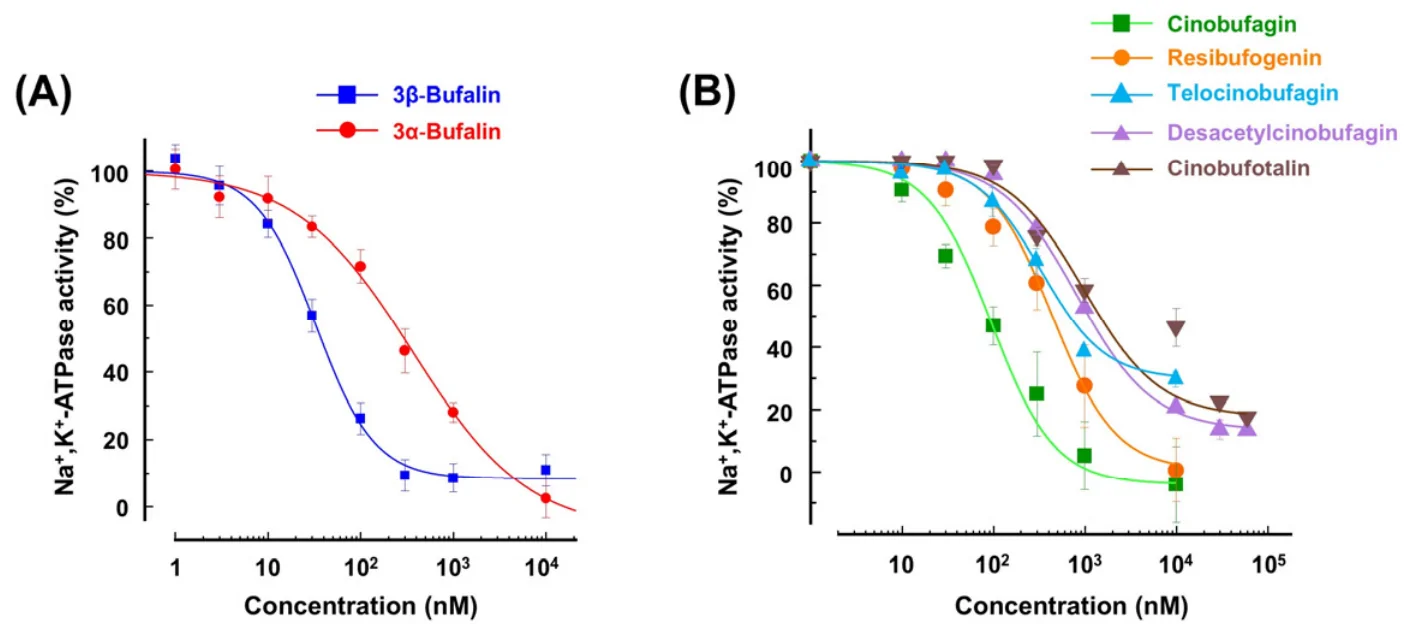
Effects of bufadienolides on Na^+^,K^+^-ATPase activity in membrane fractions of HT-29 cells. (**A**) Concentration-dependent effects of 3β-bufalin (*n* = 4) and 3α-bufalin (*n* = 5) on Na^+^,K^+^-ATPase activity in the membrane fractions. (**B**) Concentration-dependent effects of other bufadienolides, cinobufagin, resibufogenin, telocinobufagin, desacetylcinobufagin, and cinobufotalin, on Na^+^,K^+^-ATPase activity (*n* = 3). Data are presented as mean ± SEM.

Next, we examined whether the potent anti-proliferative effect of 3β-bufalin could be explained by the inhibition of Na^+^,K^+^-ATPase pumping activity. In membrane fractions prepared from HT-29 cells, 3β-bufalin inhibited Na^+^,K^+^-ATPase activity in a concentration-dependent manner, with an IC_50_ of approximately 37 nM (Figure 3A). 3α-Bufalin also inhibited Na^+^,K^+^-ATPase activity, but its potency was lower, with an IC_50_ of approximately 294 nM. Importantly, the IC_50_ of 3β-bufalin for Na^+^,K^+^-ATPase inhibition was much higher than that for proliferation inhibition in HT-29 cells. A similar tendency was also observed in KB cells, in which 3β-bufalin inhibited Na^+^,K^+^-ATPase activity with an IC_50_ of approximately 34 nM, whereas 3α-bufalin showed weaker inhibition, with an IC_50_ of approximately 249 nM (Supplementary Figure S1). In both HT-29 and KB cells, the concentrations required for 3β-bufalin to inhibit Na^+^,K^+^-ATPase activity were more than one order of magnitude higher than those required to suppress cell proliferation. These results indicate that the anti-proliferative effect of 3β-bufalin occurs at concentrations lower than those required to directly inhibit Na^+^,K^+^-ATPase pumping activity. 

We further examined the effects of other bufadienolides on Na^+^,K^+^-ATPase activity. The estimated IC_50_ values were approximately 87 nM for cinobufagin, 447 nM for resibufogenin, 708 nM for telocinobufagin, 1.1 μM for desacetylcinobufagin, and 1.5 μM for cinobufotalin (Figure 3B). Thus, all tested bufadienolides were less potent than 3β-bufalin in inhibiting Na^+^,K^+^-ATPase activity, although some showed measurable inhibition at higher concentrations.

**Figure 4:**
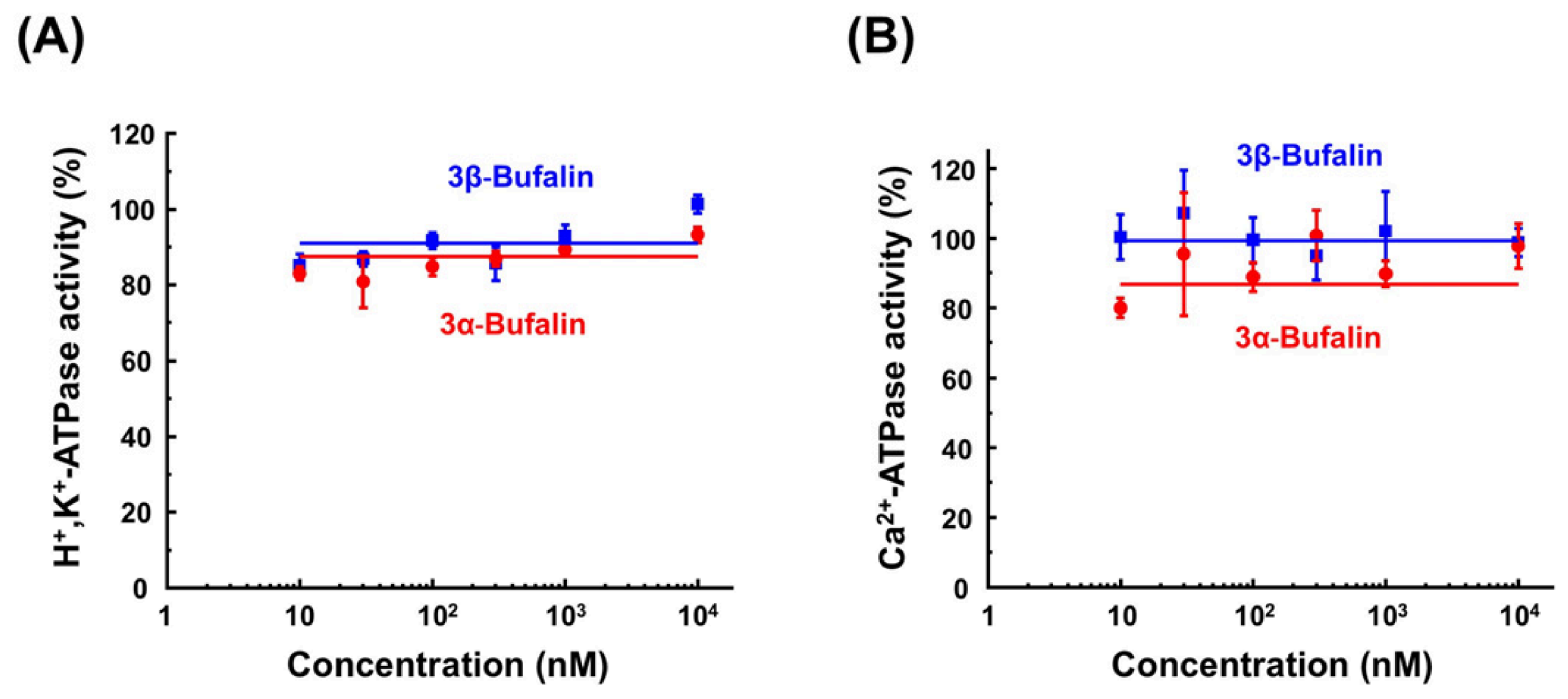
Effects of 3β-bufalin and 3α-bufalin on H^+^,K^+^-ATPase and Ca^2+^-ATPase activities. (**A**) Effects of 3β-bufalin and 3α-bufalin on hog gastric H^+^,K^+^-ATPase activity. (**B**) Effects of 3β-bufalin and 3α-bufalin on rabbit Ca^2+^-ATPase activity. Data are presented as means ± SEM; *n* = 3.

To evaluate whether bufalin preferentially targets Na^+^,K^+^-ATPase, we examined its effects on gastric H^+^,K^+^-ATPase and sarco/endoplasmic reticulum Ca^2+^-ATPase (SERCA), two cation-transporting P2-type ATPases. H^+^,K^+^-ATPase is one of the closest P2-type ATPases to Na^+^,K^+^-ATPase, and its catalytic α-subunit shares approximately 60% amino acid sequence identity with the Na^+^,K^+^-ATPase α1-isoform, whereas SERCA shares approximately 30%. H^+^,K^+^-ATPase activity was measured in hog gastric tubulovesicles, and Ca^2+^-ATPase activity in rabbit sarcoplasmic reticulum (SR) vesicles. Neither 3β-bufalin nor 3α-bufalin significantly altered H^+^,K^+^-ATPase activity or Ca^2+^-ATPase activity at concentrations of up to 10 μM (Figure 4). 

### 2.4. 3β-Bufalin, but Not 3α-Bufalin, Activates VRAC Currents in HT-29 Cells

**Figure 5:**
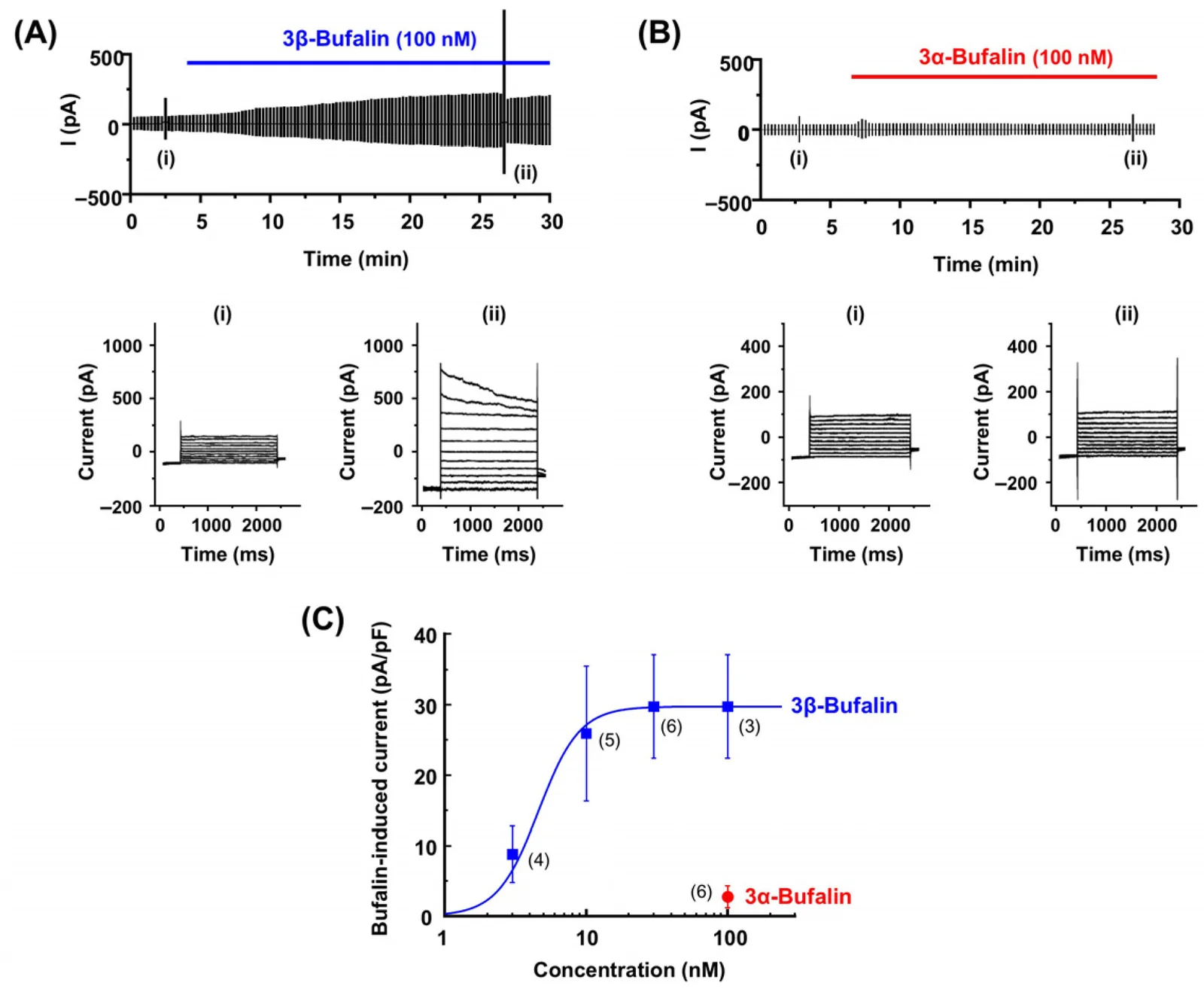
3β-Bufalin activates VRAC currents in HT-29 cells. (**A**,**B**) Upper panels: Representative whole-cell current traces at a holding potential of 0 mV in response to voltage steps to ±40 mV. 3β-Bufalin or 3α-bufalin (100 nM) was applied during the periods indicated by the blue or red lines, respectively. Step-pulse protocols were applied at the time points indicated as (**i**) and (**ii**). Lower panels: Current traces obtained in response to step pulses from −100 mV to +100 mV at (**i**) and (**ii**). (**C**) Concentration-dependent effects of 3β-bufalin and the effect of 3α-bufalin (100 nM) on the mean current density induced by a voltage pulse of +100 mV. Data are presented as means ± SEM; *n* = 3–6.

Given that 3β-bufalin suppressed cell proliferation at concentrations lower than those required to inhibit Na^+^,K^+^-ATPase activity, we examined whether the receptor-type (non-pumping) Na^+^,K^+^-ATPase in membrane microdomains of cancer cells is involved. Volume-regulated anion channel (VRAC) currents were measured to assess the involvement of receptor-type Na^+^,K^+^-ATPase signaling. Whole-cell patch-clamp recordings showed that applying 3β-bufalin (100 nM) gradually activated ion currents in HT-29 cells (Figure 5A,C). The bufalin-induced currents exhibited outward rectification with time-dependent inactivation at depolarized potentials (Figure 5A), consistent with the electrophysiological properties of VRAC currents. In contrast, 3α-bufalin failed to induce detectable VRAC activation even at 100 nM (Figure 5B). Concentration–response analysis showed an EC_50_ of 4.6 nM for 3β-bufalin-activated VRAC currents, comparable to the IC_50_ value for proliferation inhibition (Figure 5C).

We further examined whether other bufadienolides activate VRAC currents. Cinobufagin, resibufogenin, and desacetylcinobufagin did not induce detectable VRAC activation at 100 nM (Supplementary Figure S2). These results indicate that VRAC activation is not a common property of bufadienolides but is specific to 3β-bufalin among the compounds examined. Thus, the anti-proliferative potency of 3β-bufalin more closely corresponds to its ability to activate VRAC than its ability to inhibit Na^+^,K^+^-ATPase pumping activity.

### 2.5. VRAC in Membrane Microdomains Is Involved in 3β-Bufalin-Induced Anti-Proliferative Effect

**Figure 6:**
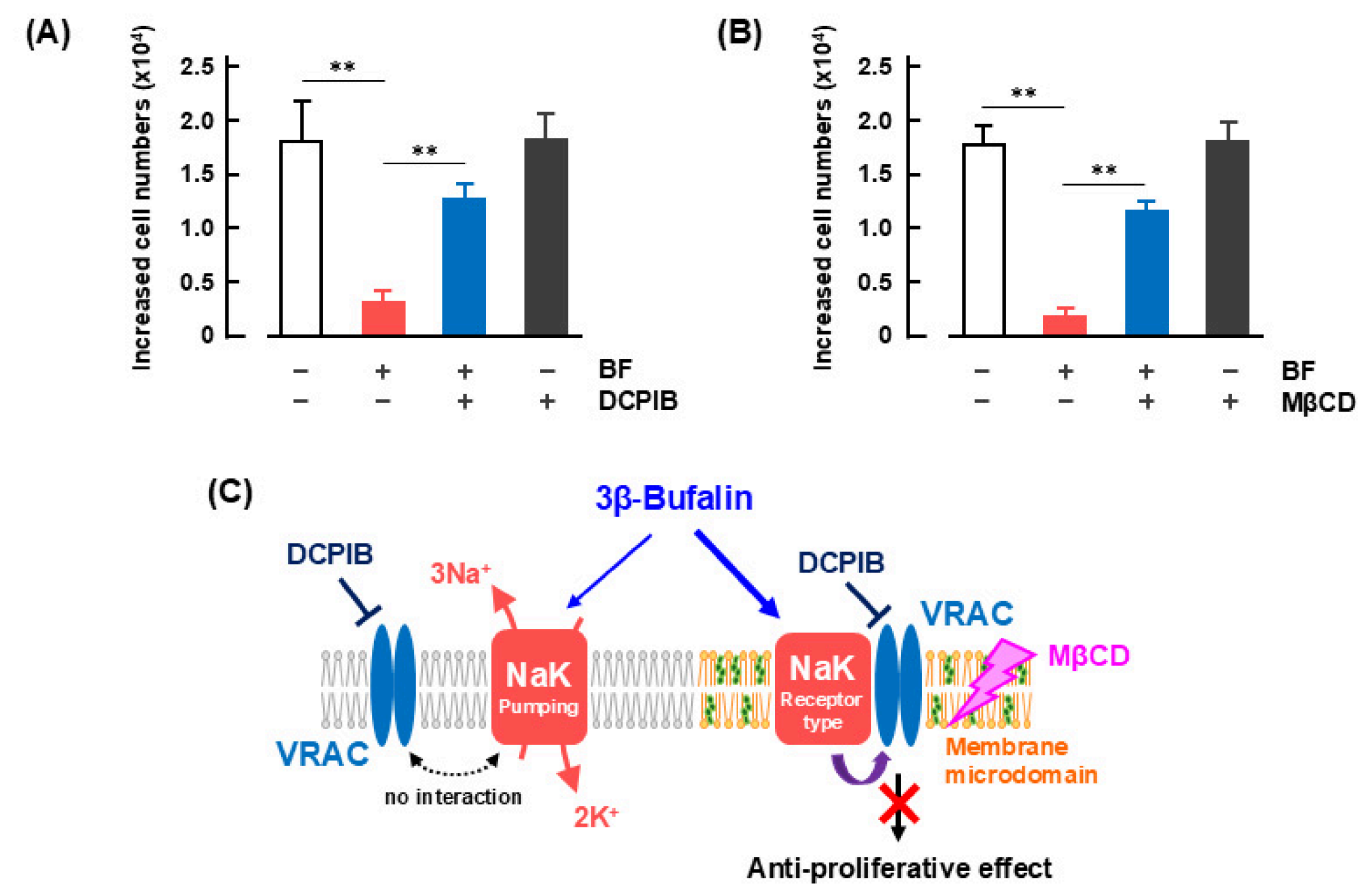
Contribution of VRAC in membrane microdomains to anti-proliferative effect of 3β-bufalin on HT-29 cells. (**A**) Effect of DCPIB (10 μM) and/or 3β-bufalin (BF; 30 nM) on cell proliferation. *n* = 5. (**B**) Effect of MβCD (10 μM) and/or 3β-bufalin (BF; 30 nM) on cell proliferation. *n* = 6. ** *p* < 0.01. (**C**) Proposed model for 3β-bufalin-induced VRAC activation in membrane microdomains. Cholesterol within membrane microdomains is indicated by green symbols.

The Na^+^,K^+^-ATPase α1-isoform is functionally coupled to the VRAC in the membrane microdomains of human cancer cells [18]. To determine whether VRAC activation in membrane microdomains is involved in the anti-proliferative effect of 3β-bufalin, we examined the effects of 4-(2-butyl-6,7-dichloro-2-cyclopentylindan-1-on-5-yl)oxybutyric acid (DCPIB), a VRAC inhibitor, and methyl-β-cyclodextrin (MβCD), a cholesterol-extracting agent that disrupts cholesterol-rich membrane microdomains, on the 3β-bufalin-induced inhibition of HT-29 cell proliferation. As shown in Figure 6A, DCPIB (10 μM) significantly decreased the 3β-bufalin (30 nM)-induced inhibition of cell proliferation, while DCPIB alone had no effect. As shown in Figure 6B, MβCD (10 μM) significantly reversed the 3β-bufalin (30 nM)-induced inhibition of cell proliferation. These results indicate that VRAC functionally coupled to receptor-type Na^+^,K^+^-ATPase in membrane microdomains is involved in the 3β-bufalin-induced anti-proliferative pathway, whereas VRAC located outside membrane microdomains, which is not coupled to pumping Na^+^,K^+^-ATPase, may not contribute to this pathway (Figure 6C).

### 2.6. VRAC Coupled to Receptor-Type Na^+^,K^+^-ATPase Is Involved in 3β-Bufalin-Induced G2/M Cell Cycle Arrest

**Figure 7:**
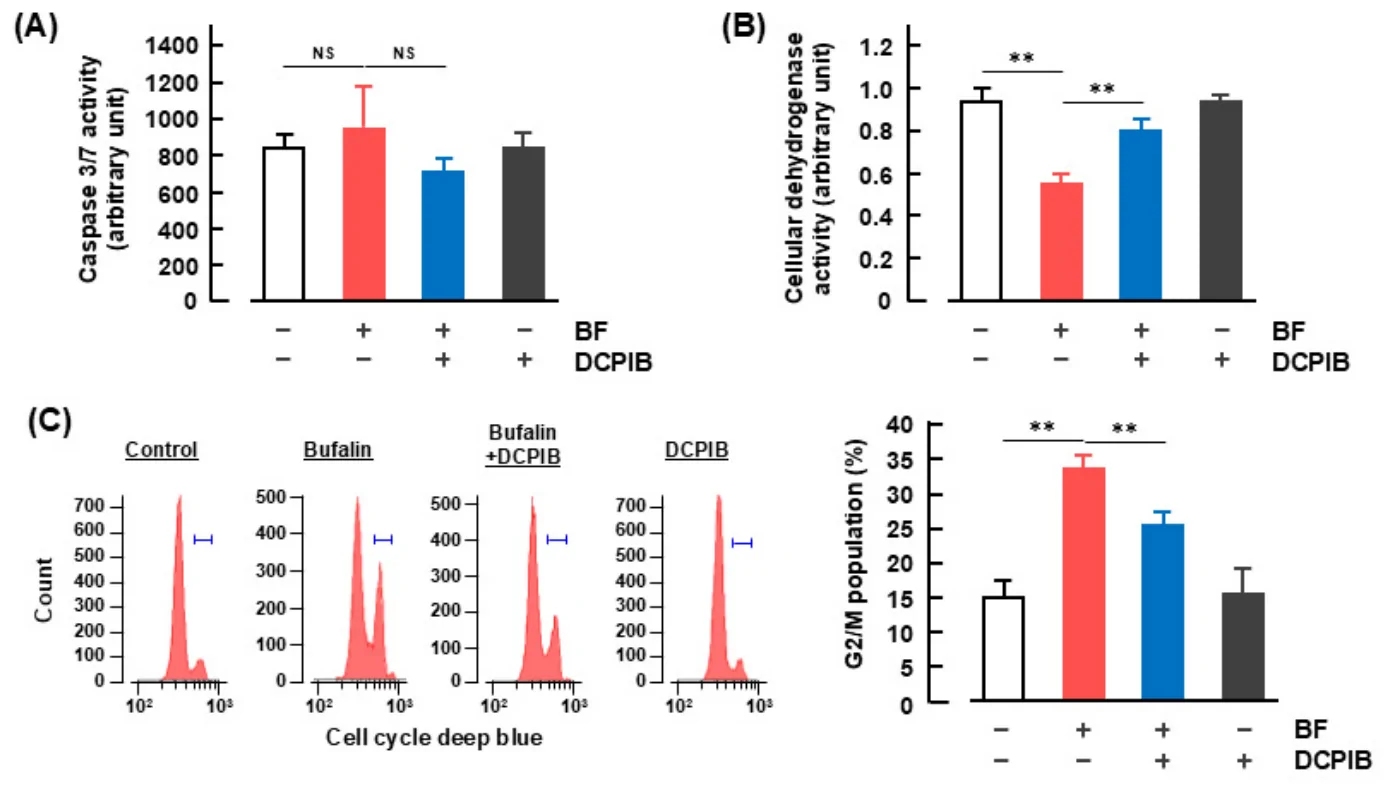
Elucidation of 3β-bufalin-induced cellular responses mediated by receptor-type Na^+^,K^+^-ATPase and VRAC in HT-29 cells. (**A**) Effects of 3β-bufalin (BF; 30 nM) and/or DCPIB (10 μM) on caspase-3/7 activity in cells. *n* = 4. (**B**) Effects of 3β-bufalin (BF; 30 nM) and/or DCPIB (10 μM) on cellular dehydrogenase activity in cells. *n* = 4. (**C**) Effects of 3β-bufalin (BF; 30 nM) and/or DCPIB (10 μM) on cell cycle. Representative flow cytometric profiles of cell cycle distribution (left) and quantitative analysis of proportion of cells in G2/M phase (right). The G2/M region is indicated by the blue bracket. *n* = 3. Data are presented as means ± SEM. ** *p* < 0.01; NS, not significant.

To further characterize the cellular mechanism underlying the anti-proliferative effect of 3β-bufalin, we examined caspase-3/7 activity, cellular dehydrogenase activity, and cell cycle distribution. Treatment with 3β-bufalin (30 nM) did not significantly increase caspase-3/7 activity (Figure 7A), but significantly reduced cellular dehydrogenase activity, which was significantly recovered under DCPIB treatment (10 μM) (Figure 7B). Furthermore, 3β-bufalin significantly increased the proportion of cells in the G2/M phase, an effect significantly attenuated by DCPIB (Figure 7C). These results indicate that VRAC activation in membrane microdomains contributes to 3β-bufalin-induced alterations in cellular dehydrogenase activity and G2/M cell cycle progression, rather than substantially inducing caspase-dependent apoptosis.

## 3. Discussion

**Figure 8:**
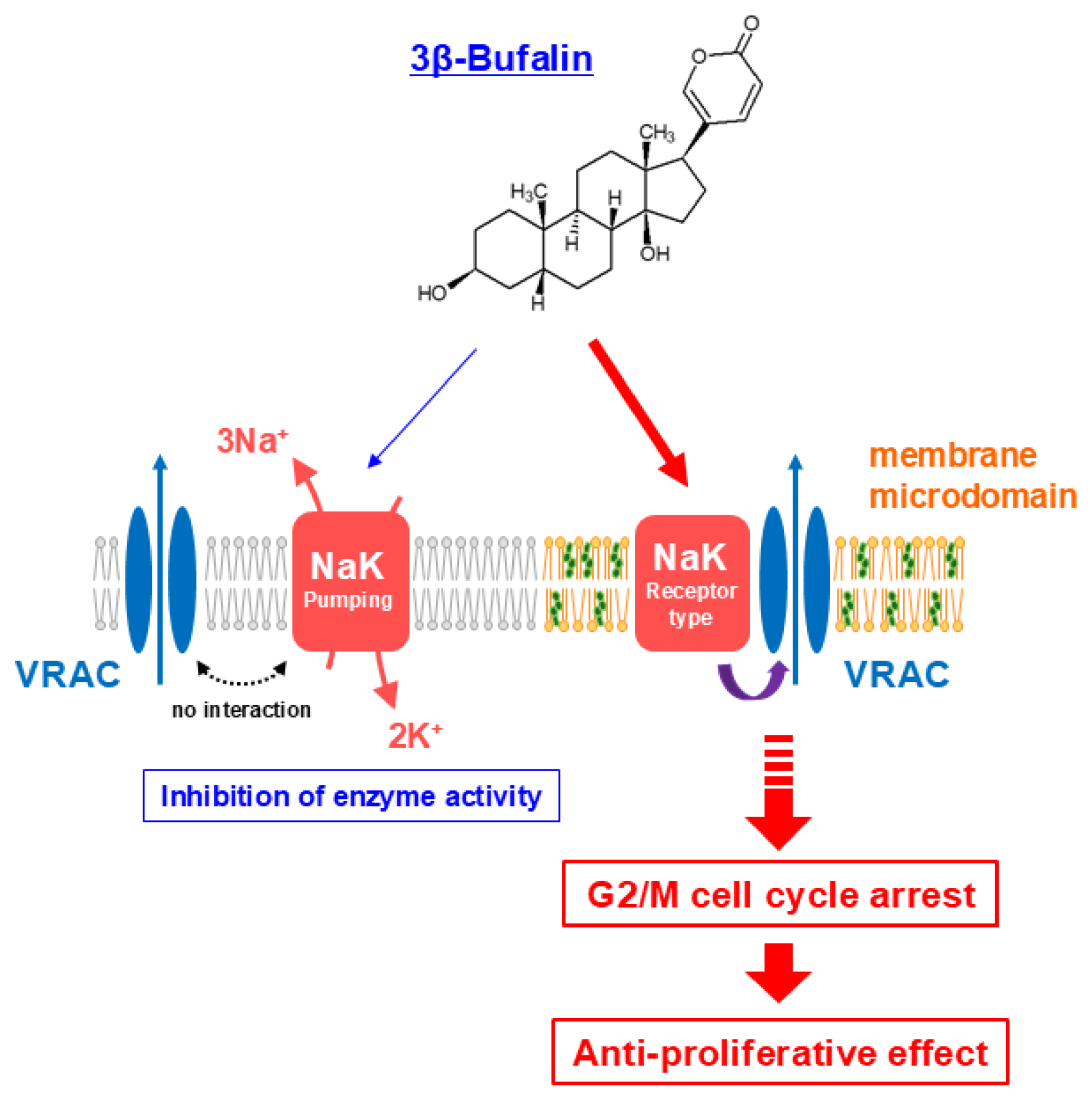
Proposed mechanism of 3β-bufalin-induced anti-proliferative action through receptor-type Na^+^,K^+^-ATPase-mediated VRAC activation. At relatively higher concentrations, 3β-bufalin inhibits the ion-pumping activity of Na^+^,K^+^-ATPase (blue arrow). At low nanomolar concentrations, 3β-bufalin is proposed to act on receptor-type, non-pumping Na^+^,K^+^-ATPase in membrane microdomains (red arrow), activating VRAC. VRAC activation induces G2/M cell cycle arrest, thereby suppressing cancer cell proliferation. Cholesterol within membrane microdomains is indicated by green symbols.

The present study was designed to clarify how toad-derived bufadienolides act on Na^+^,K^+^-ATPase-mediated anti-cancer mechanisms, with particular attention to the distinction between the inhibition of ion-pumping activity and the induction of receptor-type signaling. The main finding was that among the tested bufadienolides, 3β-bufalin exhibited a distinctive pattern of activity: it suppressed cancer cell proliferation at low nanomolar concentrations, showed strong dependence on C3 hydroxyl stereochemistry, and activated the VRAC at concentrations close to those required to suppress growth. In contrast, inhibiting Na^+^,K^+^-ATPase activity required higher concentrations than those needed to suppress growth, and VRAC activation was observed with 3β-bufalin but not with 3α-bufalin or the other tested bufadienolides. Thus, VRAC activation is not a common property of all bufadienolides but appears to be selectively associated with the potent anti-proliferative activity of 3β-bufalin. Importantly, DCPIB-induced pharmacological inhibition of the VRAC and MβCD-induced disruption of cholesterol-rich membrane microdomains markedly attenuated the anti-proliferative effect of 3β-bufalin. These findings support the hypothesis that the anti-proliferative action of 3β-bufalin is associated with VRAC activation through functional coupling with receptor-type Na^+^,K^+^-ATPase within membrane microdomains, rather than direct inhibition of Na^+^,K^+^-ATPase pumping activity (Figure 8). 

Previous structure–activity relationship studies have shown that structural modifications of bufadienolides, including hydroxylation, acetylation, epoxidation, glycosylation, and modifications at C3, affect their cytotoxicity, Na^+^,K^+^-ATPase inhibitory activity, and interactions with Na^+^,K^+^-ATPase [21,22,23,24,25,26,27,28]. Such studies provide an important basis for interpreting the pharmacological properties of bufadienolides; however, they mainly addressed the cytotoxicity, toxicity, or inhibition of Na^+^,K^+^-ATPase pumping activity. In the present study, the conversion of 3β-bufalin into 3α-bufalin markedly reduced not only Na^+^,K^+^-ATPase inhibition but also anti-proliferative activity and VRAC activation. VRAC activation occurred at concentrations close to those required to suppress growth and lower than those required to inhibit Na^+^,K^+^-ATPase activity. Moreover, VRAC inhibition and the disruption of membrane microdomains attenuated the anti-proliferative effect of 3β-bufalin. Therefore, C3 stereochemistry appears to be important for receptor-type Na^+^,K^+^-ATPase signaling, as well as for the previously reported pump inhibition and cytotoxicity of bufadienolides. 

The comparison with other bufadienolides further indicates that 1β-hydroxylation and 14β,15β-epoxidation modulate the functional profile of these compounds. Telocinobufagin differs from 3β-bufalin mainly due to an additional 1β-hydroxyl group and lower potency in inhibiting Na^+^,K^+^-ATPase activity, as shown here. A similar relationship was observed between cinobufagin and cinobufotalin, where the latter presents an additional 1β-hydroxyl group. In addition, cinobufagin and resibufogenin contain a 14β,15β-epoxide moiety, whereas 3β-bufalin contains a 14β-hydroxyl group. Previous work using microbial hydroxylation products of bufalin reported that the introduction of a 14β,15β-epoxide reduced both Na^+^,K^+^-ATPase inhibitory activity and cytotoxicity [29]. In the present study, although cinobufagin retained moderate Na^+^,K^+^-ATPase inhibition, neither cinobufagin nor resibufogenin activated the VRAC under the tested conditions. These observations indicate that 14β,15β-epoxidation reduces Na^+^,K^+^-ATPase inhibition and cytotoxicity in cancer cells and may also be unfavorable for the conformational or microdomain-dependent events required to activate VRACs through receptor-type Na^+^,K^+^-ATPase.

Our findings indicate that the structural requirements to inhibit Na^+^,K^+^-ATPase pumping activity and activate VRAC signaling through receptor-type Na^+^,K^+^-ATPase partially overlap but are not identical in cancer cells. Crystal structure analyses have provided important information on the binding modes of cardiotonic steroids to Na^+^,K^+^-ATPase. Studies on bufalin-bound Na^+^,K^+^-ATPase structures showed that bufalin binds in the transmembrane region and determined the structural features involved in pump inhibition [30,31]. However, such structural studies were performed under purified or crystallized conditions, making it difficult to directly compare bufalin binding in these structures with receptor-type, non-pumping Na^+^,K^+^-ATPase organized in membrane microdomains of living cancer cells. Receptor-type Na^+^,K^+^-ATPase may differ from ion-pumping Na^+^,K^+^-ATPase in apparent affinity for cardiotonic steroids, possibly because of differences in associated proteins, local lipid composition, cholesterol-rich membrane microdomains, or conformational constraints. Such microenvironment-dependent differences may help explain why 3β-bufalin activates the VRAC and suppresses proliferation at concentrations lower than those required to inhibit total Na^+^,K^+^-ATPase activity.

Previous studies have reported anti-cancer effects of bufalin on colorectal cancer cells at nanomolar to submicromolar concentrations. 3β-Bufalin has been shown to suppress cancer cell growth and induce cell death through several signaling pathways, including JNK activation [32], hTERT-related pathways [33], C-Kit/Slug signaling [34], and mitochondrial ROS-mediated caspase-3 activation [35]. Bufalin has also been reported to induce G2/M cell cycle arrest in colon cancers, including HT-29 cells [36,37,38]. In the present study, treatment with 3β-bufalin at 30 nM did not significantly increase caspase-3/7 activity in HT-29 cells, whereas it markedly increased the proportion of cells in the G2/M phase. Notably, the increase in the G2/M population was significantly attenuated by DCPIB, which indicates that VRAC activity contributes to 3β-bufalin-induced cell cycle arrest. VRAC inhibition also mitigated the 3β-bufalin-induced reduction in cellular dehydrogenase activity, indicating an association between this metabolic change and G2/M arrest. Further studies are warranted to elucidate the molecular mechanisms linking VRAC activation to these cellular responses. 

To further assess the pharmacokinetic and therapeutic potential of bufadienolides, we performed a preliminary in silico ADME analysis using SwissADME (Supplementary Table S1). High gastrointestinal absorption and blood–brain barrier permeability were predicted for 3β-bufalin, consistent with its reported brain penetration in mice [39]. 3β-Bufalin was also predicted to be a P-glycoprotein substrate and showed favorable drug-likeness, whereas inhibition of major CYP isoforms, including CYP3A4, was not predicted. However, previous studies have shown that bufalin is metabolized by CYP3A and can inhibit CYP3A activity [40,41,42], indicating that in silico predictions do not fully capture its experimentally determined pharmacokinetic properties. Although SwissADME predicted broadly similar ADME-related profiles for 3β-bufalin and 3α-bufalin, such predictions may not fully account for stereochemistry-dependent differences in metabolism, transporter interactions, or tissue distribution. In this regard, a previous pharmacokinetic study in rats reported that after oral administration of bufalin, 3α-bufalin was detected as the major circulating metabolite, whereas unchanged bufalin was detected only at the highest dose tested [20]. Nevertheless, the pharmacokinetic profiles of independently administered 3β-bufalin and 3α-bufalin have not been directly compared. Further experimental pharmacokinetic studies are therefore required to better define the therapeutic potential of 3β-bufalin and to clarify how the stereochemical configuration of the C3 hydroxyl group influences the pharmacokinetic and biological properties of these bufadienolides.

## 4. Materials and Methods

### 4.1. Toad-Derived Bufadienolides and Reagents

3β-Bufalin, 3α-bufalin, resibufogenin, cinobufagin, telocinobufagin, cinobufotalin, and desacetylcinobufagin were kindly provided by Kyushin Pharmaceutical Co., Ltd. (Tokyo, Japan). The purity of each compound was determined with high-performance liquid chromatography (HPLC): 3β-bufalin, 99.5%; 3α-bufalin, 98.8%; resibufogenin, 99.7%; cinobufagin, 99.3%; telocinobufagin, 94.5%; cinobufotalin, 97.0%; and desacetylcinobufagin, 99.4%. The corresponding HPLC chromatograms are shown in Supplementary Figure S3. Each bufadienolide was dissolved in dimethyl sulfoxide (DMSO) to prepare stock solutions. DCPIB and MβCD were obtained from Tocris Bioscience (Bristol, UK) and Sigma-Aldrich (St. Louis, MO, USA), respectively. All other reagents were of molecular biology grade or the highest purity available.

### 4.2. Cell Culture

Human colorectal cancer HT-29 cells (ATCC, Cat. No. HTB-38) were maintained in Dulbecco’s Modified Eagle’s medium (DMEM; Fujifilm Wako, Osaka, Japan) supplemented with 100 units/mL penicillin, 100 µg/mL streptomycin (Fujifilm Wako), and 10% fetal bovine serum (FBS; Nichirei Biosciences, Tokyo, Japan) under conditions of 37 °C and 5% CO_2_. Human oral cancer KB cells (kindly gifted by Prof. Yasunobu Okada, National Institute for Physiological Sciences) were maintained in minimum essential medium (MEM; Fujifilm Wako) supplemented with 100 units/mL penicillin, 100 µg/mL streptomycin, and 10% FBS under conditions of 37 °C and 5% CO_2_. Cells were passaged before reaching confluence.

### 4.3. Cell Proliferation Assay

Cell proliferation was measured as previously described [43]. In each well of a 96-well culture plate, 2 × 10^4^ cells were seeded. After seeding (24 h later), the number of cells in each well was counted (first count). The cells were then treated with or without each bufadienolide at 30 nM for another 24 h in the culture medium supplemented with 2% FBS. The number of cells in each well was counted after the culture (second count). Cell proliferation was evaluated by quantifying the increase in cell number between the first and second counts.

### 4.4. Preparation of Cell Membrane Fractions

Membrane fractions were prepared from HT-29 and KB cells, as described previously [44]. The cells were resuspended and homogenized in a solution containing 0.5 mM MgCl_2_ and 10 mM Tris-HCl (pH 7.4). The homogenates were centrifuged at 500× *g* for 10 min at 4 °C, and the resulting supernatants were centrifuged at 100,000× *g* for 90 min at 4 °C. The membrane pellets were resuspended in a solution containing 250 mM sucrose and 5 mM Tris-HCl (pH 7.4) and used as membrane fractions. Protein concentrations were determined using the BCA Protein Assay Kit (Thermo Fisher Scientific, Rockford, IL, USA) with BSA as the standard by measuring the absorbance at 570 nm (Sunrise Remote microplate reader, Tecan, Männedorf, Switzerland).

### 4.5. Measurement of Na^+^,K^+^-ATPase Activity

Na^+^,K^+^-ATPase activity was measured in membrane fractions prepared from HT-29 and KB cells, as described previously [7]. Membrane fractions (50 μg of protein) were incubated in a reaction solution containing 120 mM NaCl, 15 mM KCl, 3 mM MgSO_4_, 1 mM ATP, 5 mM NaN_3_, and 10 mM HEPES-Tris (pH 7.4) in the absence or presence of the indicated concentrations of bufadienolides. After incubation for 30 min at 37 °C, the reaction was terminated by adding an ice-cold stop solution containing 12% perchloric acid and 3.6% ammonium molybdate. 

### 4.6. Measurement of H^+^,K^+^-ATPase and Ca^2+^-ATPase Activity

H^+^,K^+^-ATPase activity was measured using membrane samples prepared from hog gastric tubulovesicles, as described previously [45]. Briefly, gastric mucosa was scraped from hog stomachs, minced, and homogenized in a solution containing 250 mM sucrose, 1 mM EGTA, and 5 mM Tris-HCl (pH 7.4). The homogenate was centrifuged at 1000× *g* for 10 min, and the resulting supernatant was centrifuged at 13,500× *g* for 30 min. The supernatant was then centrifuged at 100,000× *g* for 30 min. The pellet obtained after ultracentrifugation was resuspended in 250 mM sucrose, homogenized, layered onto a step gradient consisting of 250 mM sucrose and 7% Ficoll, and centrifuged at 132,000× *g* for 1 h. Light vesicles collected from the interface between the 250 mM sucrose and 7% Ficoll layers were used as tubulovesicles. Freeze-dried tubulovesicles (7.5 μg of protein) were incubated in 250 μL of reaction solution containing 3 mM MgSO_4_, 1 mM ATP, 15 mM KCl, 5 mM NaN_3_, 100 μM ouabain, and 40 mM Tris-HCl (pH 6.8), in the absence or presence of 50 μM SCH28080, a selective inhibitor of gastric H^+^,K^+^-ATPase. After incubation for 10 min at 37 °C, the reaction was terminated by adding an ice-cold stop solution containing 12% perchloric acid and 3.6% ammonium molybdate. The released inorganic phosphate was measured, and H^+^,K^+^-ATPase activity was calculated as the SCH28080-sensitive component of ATPase activity. 

Ca^2+^-ATPase activity was measured using a microsomal fraction enriched in the transverse tubular system (T-tubule)/sarcoplasmic reticulum (SR) complex prepared from rabbit back and leg muscles, as described previously [46,47]. The T-tubule/SR complex (5 μg of protein) was incubated in 1 mL of reaction solution containing 100 mM KCl, 7 mM MgCl_2_, 2 mM CaCl_2_, 2 mM EGTA, 1 mM ATP, 1 μM A23187, and 50 mM MOPS-Tris (pH 7.0), in the presence or absence of 100 nM thapsigargin. The free Ca^2+^ concentration of the solution was estimated to be 20 μM. After incubation for 10 min at 37 °C, the reaction was terminated by adding ice-cold stop solution, and the released inorganic phosphate was measured. Ca^2+^-ATPase activity was calculated as the difference in ATPase activity measured in the absence and presence of 100 nM thapsigargin.

### 4.7. Whole-Cell Patch-Clamp Recording

Whole-cell patch-clamp recordings were performed to measure VRAC currents in HT-29 cells, as previously described with minor modifications [18]. Recordings were performed using an EPC-10 patch-clamp amplifier (HEKA Elektronik, Reutlingen, Germany). PatchMaster software (HEKA Elektronik) was used for command pulse control and data acquisition. Data were filtered at 2.9 kHz and digitized at 10 kHz. The acquired data were analyzed using WinASCD software, kindly provided by Prof. G. Droogmans, and Clampfit 10.6 software (Molecular Devices, LLC, San Jose, CA, USA). Patch electrodes had a resistance of 2–4 MΩ when filled with pipette solution. Access resistance was electrically compensated to 70% to minimize voltage errors. Current–voltage relationships were obtained from currents elicited with voltage-step pulses ranging from −100 to +100 mV in 20-mV increments with a pre-potential of −100 mV and a post-potential of −60 mV. Instantaneous currents were measured at +100 mV during the step pulses. Steady-state currents were averaged between 450 and 500 ms during the step pulses. Currents were normalized to the corresponding membrane capacitance. The pipette solution contained 110 mM CsCl, 2 mM MgSO_4_, 1 mM Na_2_ATP, 1 mM EGTA, 10 mM HEPES, 15 mM Na-HEPES, and 50 mM mannitol (pH 7.3). The control bathing solution (305 mOsmol/kg H_2_O) contained 110 mM CsCl, 5 mM MgSO_4_, 7 mM Tris, 12 mM HEPES, and 75 mM mannitol (pH 7.5). 

### 4.8. Measurement of Caspase 3/7 Activity

HT-29 cells cultured in 6-well plates were detached using 0.25% trypsin–EDTA after treatment with the indicated compounds and were counted. Caspase-3/7 activity was measured using the Caspase-Glo 3/7 Assay (Promega, Madison, WI, USA). A cell suspension containing 1 × 10^5^ cells in 50 μL was mixed with 50 μL of Caspase-Glo 3/7 reagent and incubated at room temperature for 1 h. Luminescence was measured using a FilterMax F5 Multi-Mode Microplate Reader (Molecular Devices, LLC, San Jose, CA, USA).

### 4.9. Measurement of Cellular Dehydrogenase Activity

HT-29 cells cultured in 6-well plates were detached using 0.25% trypsin–EDTA after treatment with the indicated compounds and counted. An equal number of cells (1 × 10^4^ cells) was suspended in 100 μL of culture medium and transferred to each well of a 96-well plate. Cellular dehydrogenase activity was measured using Cell Counting Kit-8 (Dojindo Laboratories, Kumamoto, Japan). Cell Counting Kit-8 solution (10 μL) was added to each well, and the cells were incubated at 37 °C for 1 h. Absorbance at 450 nm was then measured using a FilterMax F5 Multi-Mode Microplate Reader.

### 4.10. Cell Cycle Analysis

HT-29 cells cultured in 6-well plates were detached using 0.25% trypsin–EDTA and washed three times with PBS by centrifuging them at 200× *g* for 5 min. The cells were counted and adjusted to a density of 5 × 10^5^ cells/mL. For cell cycle analysis, 5 μL of Cell Cycle Assay Solution Blue (Dojindo Laboratories) was added to 500 μL of the cell suspension. The cells were incubated at 37 °C for 15 min in the dark and subsequently analyzed using a BD FACSymphony A1 flow cytometer (BD Biosciences, San Jose, CA, USA) with excitation at 405 nm, and fluorescence was detected using a 450/50 nm bandpass filter. 

### 4.11. Statistical Analysis

Data are presented as means ± SEM unless otherwise indicated. Statistical analyses were performed using the appropriate tests for each experimental design. Comparisons between two groups were performed using Student’s *t*-test, and comparisons among multiple groups were performed using one-way analysis of variance, followed by a suitable post hoc test. Concentration–response curves were fitted using nonlinear regression analysis to calculate the IC_50_ and EC_50_ values.

### 4.12. Figure Preparation

Graphs were generated using Origin 2019 (OriginLab, Northampton, MA, USA) and Microsoft Excel (Microsoft 365; Microsoft Corporation, Redmond, WA, USA), and figures and schematic illustrations were prepared using Microsoft PowerPoint (Microsoft 365).

## 5. Conclusions

Among the tested toad-derived bufadienolides, 3β-bufalin most potently suppressed cancer cell proliferation through receptor-type Na^+^,K^+^-ATPase-VRAC signaling in membrane microdomains. VRAC activation by 3β-bufalin induces G2/M cell cycle arrest, thereby inhibiting cancer cell proliferation. These findings provide mechanistic insights into the strong anti-cancer activity of 3β-bufalin, underscore the importance of C3 hydroxyl stereochemistry in bufadienolide-mediated Na^+^,K^+^-ATPase signaling, and indicate that bufadienolides and cardenolides may share a common anti-cancer pathway involving Na^+^,K^+^-ATPase–VRAC coupling in cancer cell membrane microdomains.

## Data Availability

The original contributions presented in this study are included in the article/Supplementary Materials. Further inquiries can be directed to the corresponding authors.

## Supplementary Materials

The following supporting information can be downloaded at: https://www.mdpi.com/article/10.3390/ijms27177969/s1.

## Abbreviations

The following abbreviations are used in this manuscript:

DCPIB	4-(2-butyl-6,7-dichloro-2-cyclopentylindan-1-on-5-yl)oxybutyric acid
DMEM	Dulbecco’s Modified Eagle’s Medium
DMSO	dimethyl sulfoxide
FBS	fetal bovine serum
MβCD	methyl-β-cyclodextrin
MEM	minimum essential medium
ROS	reactive oxygen species
SERCA	sarco/endoplasmic reticulum Ca^2+^-ATPase
SR	sarcoplasmic reticulum
VRAC	volume-regulated anion channel
