# Supplementary material for: Cardiac Glycoside 3β-Bufalin Suppresses Cancer Cell Proliferation by Coupling with Na+,K+-ATPase and Volume-Regulated Anion Channel Within Membrane Microdomains
Source: Int J Mol Sci. 2026 Sep 7;27(17):7969. doi: 10.3390/ijms27177969 (PMC13565899; doi:10.3390/ijms27177969)
Supplement: Supplementary file 1 [file ijms-27-07969-s001.zip › ijms-4360606-supplementary.pdf]

International Journal of
Molecular Sciences

Appendix

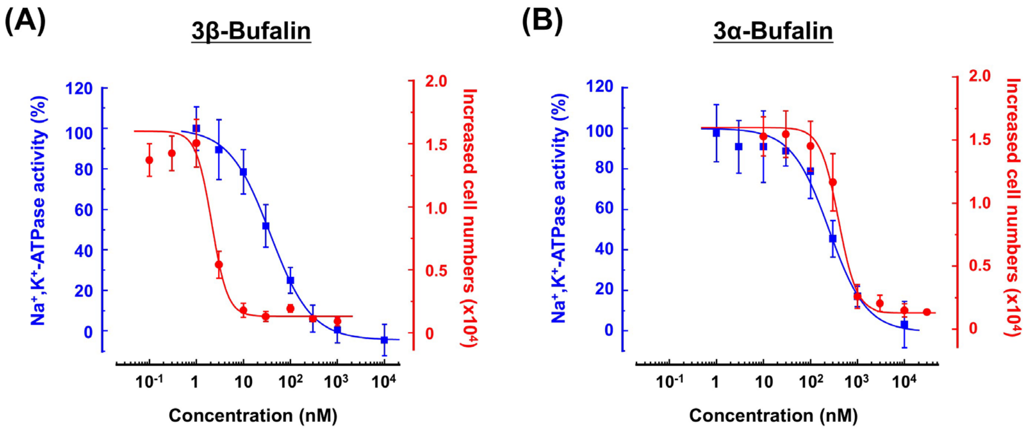

Supplementary Figure S1. Effects of 3β-bufalin and 3α-bufalin on KB cell proliferation and Na^+,K^+-ATPase activity. (A) Concentration-dependent effects of 3β-bufalin on Na^+,K^+-ATPase activity (blue) and cell proliferation (red) in human oral cancer KB cells. 3β-Bufalin suppressed KB cell proliferation, with an IC_{50} of 2.3 nM, whereas it inhibited Na^+,K^+-ATPase activity, with an IC_{50} of approximately 34 nM. (B) Concentration-dependent effects of 3α-bufalin on Na^+,K^+-ATPase activity (blue) and cell proliferation (red) in human oral cancer KB cells. 3α-Bufalin suppressed KB cell proliferation, with an IC_{50} of 430 nM, whereas it inhibited Na^+,K^+-ATPase activity, with an IC_{50} of approximately 249 nM. Data are presented as means ± SEM.

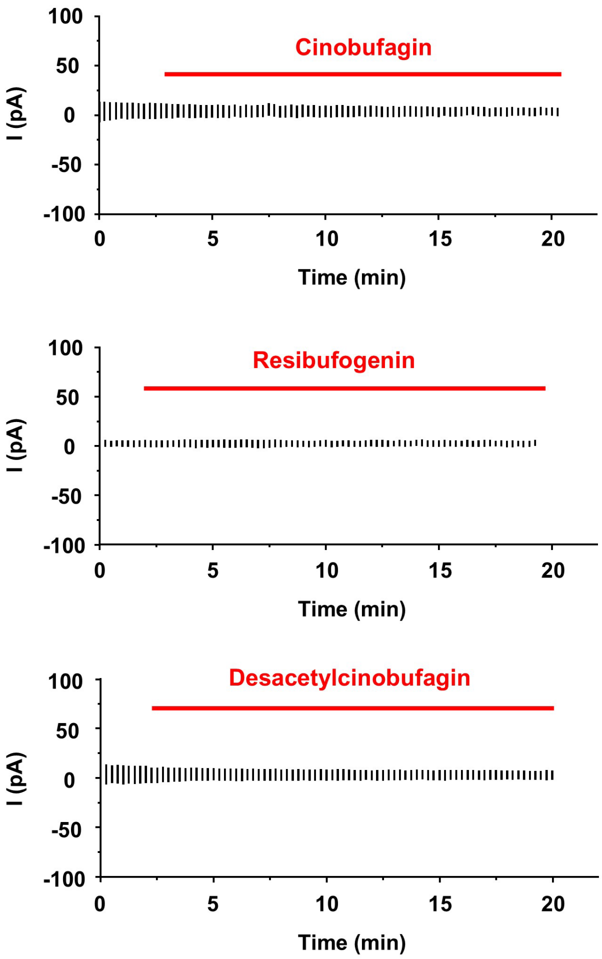

Supplementary Figure S2. Effects of other bufadienolides on VRAC currents in HT-29 cells. Whole-cell patch-clamp recordings were performed to determine whether cinobufagin, resibufogenin, and desacetylcinobufagin activate VRAC currents in HT-29 cells.

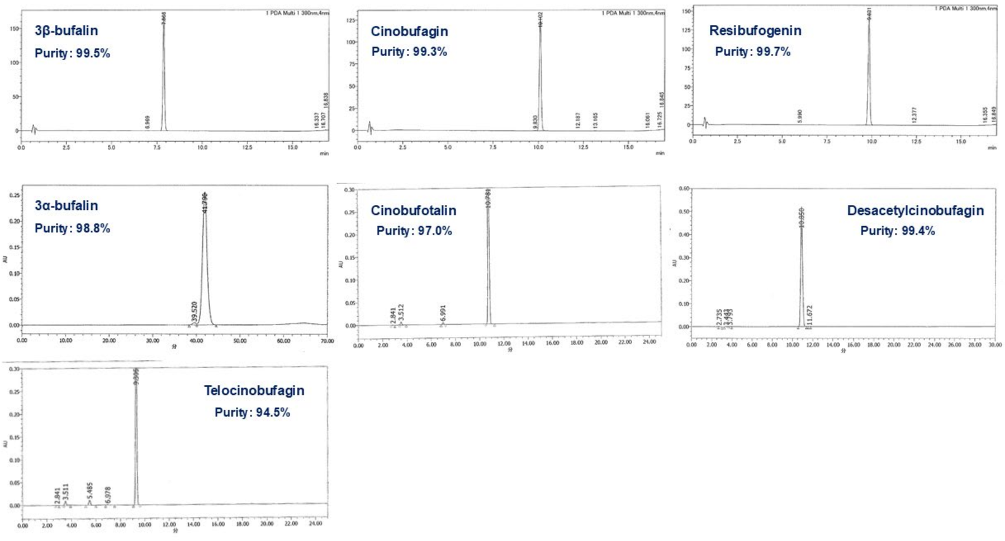

Supplementary Figure S3. HPLC chromatograms of the bufadienolides used in this study. Representative HPLC chromatograms of 3β-bufalin, cinobufagin, resibufogenin, 3α-bufalin, cinobufotalin, desacetylcinobufagin, and telocinobufagin are shown. The purities of the compounds were 99.5%, 99.3%, 99.7%, 98.8%, 97.0%, 99.4%, and 94.5%, respectively.

Molecule	Physicochemical Properties										Lipophilicity					
	Formula	MW	#Heavy atoms	#Aromatic heavy atoms	Fraction Csp3	#Rotatable bonds	#H-bond acceptors	#H-bond donors	MR	TPSA	iLOGP	XLOGP3	WLOGP	MLOGP	Silicos-IT Log P	Consensus Log P
**3b-bufalin**	C24H34O4	386.52	28	6	0.79	1	4	2	109.86	70.67	n/d	0.35	4.24	3.58	2.37	2.64
**3a-bufalin**	C24H34O4	386.52	28	6	0.79	1	4	2	109.86	70.67	n/d	0.35	4.24	3.58	2.37	2.64
**Cinobufagin**	C26H34O6	442.54	32	6	0.77	3	6	1	118.56	89.27	n/d	0.48	3.8	3.08	2.28	2.41
**Cinobufotalin**	C26H34O7	458.54	33	6	0.77	3	7	2	119.76	109.5	n/d	-0.76	2.91	2.28	1.78	1.55
**Resibufogenin**	C24H32O4	384.51	28	6	0.79	1	4	1	107.66	62.97	n/d	0.89	4.26	3.58	2.71	2.86
**Desacetylcinobufagin**	C24H32O5	400.51	29	6	0.79	1	5	2	108.83	83.2	n/d	-0.09	3.23	2.75	1.81	1.92
**Telocinobufagin**	C24H34O5	402.52	29	6	0.79	1	5	3	111.06	90.9	n/d	-0.38	3.36	2.75	1.86	1.9

Molecule	Water solubility											
	ESOL Log S	ESOL Solubility (mg/ml)	ESOL Solubility (mol/l)	ESOL Class	Ali Log S	Ali Solubility (mg/ml)	Ali Solubility (mol/l)	Ali Class	Silicos-IT LogSw	Silicos-IT Solubility (mg/ml)	Silicos-IT Solubility (mol/l)	Silicos-IT class
**3b-bufalin**	-2.55	1.09	2.82E-03	Soluble	-1.4	15.4	4.00E-02	Very soluble	-3.58	1.01E-01	2.61E-04	Soluble
**3a-bufalin**	-2.55	1.09	2.82E-03	Soluble	-1.4	15.4	4.00E-02	Very soluble	-3.58	1.01E-01	2.61E-04	Soluble
**Cinobufagin**	-2.83	0.659	1.49E-03	Soluble	-1.92	5.27	1.19E-02	Very soluble	-3.46	1.55E-01	3.49E-04	Soluble
**Cinobufotalin**	-2.14	3.32	7.23E-03	Soluble	-1.06	39.7	8.67E-02	Very soluble	-3.08	3.77E-01	8.22E-04	Soluble
**Resibufogenin**	-2.88	0.51	1.33E-03	Soluble	-1.8	6.13	1.60E-02	Very soluble	-3.66	8.35E-02	2.17E-04	Soluble
**Desacetylcinobufagin**	-2.35	1.77	4.43E-03	Soluble	-1.21	25	6.24E-02	Very soluble	-2.84	5.74E-01	1.43E-03	Soluble
**Telocinobufagin**	-2.18	2.64	6.56E-03	Soluble	-1.07	34.6	8.59E-02	Very soluble	-3.21	2.45E-01	6.10E-04	Soluble

Molecule	Pharmacokinetics								
	GI absorption	BBB permeant	Pgp substrate	CYP1A2 inhibitor	CYP2C19 inhibitor	CYP2C9 inhibitor	CYP2D6 inhibitor	CYP3A4 inhibitor	log Kp (cm/s)
**3b-bufalin**	High	Yes	Yes	n/d	n/d	n/d	n/d	n/d	-8.41
**3a-bufalin**	High	Yes	Yes	n/d	n/d	n/d	n/d	n/d	-8.41
**Cinobufagin**	High	No	Yes	n/d	n/d	n/d	n/d	n/d	-8.66
**Cinobufotalin**	High	No	Yes	n/d	n/d	n/d	n/d	n/d	-9.64
**Resibufogenin**	High	Yes	Yes	n/d	n/d	n/d	n/d	n/d	-8.01
**Desacetylcinobufagin**	High	No	Yes	n/d	n/d	n/d	n/d	n/d	-8.81
**Telocinobufagin**	High	No	Yes	n/d	n/d	n/d	n/d	n/d	-9.03

Molecule	Druglikeness						Medicinal Chemistry			
	Lipinski #violations	Ghose #violations	Veber #violations	Egan #violations	Muegge #violations	Bioavailability Score	PAINS #alerts	Brenk #alerts	Leadlikeness #violations	Synthetic Accessibility
**3b-bufalin**	0	0	0	0	0	0.55	0	0	1	5.56
**3a-bufalin**	0	0	0	0	0	0.55	0	0	1	5.56
**Cinobufagin**	0	0	0	0	0	0.55	0	1	1	6.48
**Cinobufotalin**	0	0	0	0	0	0.55	0	1	1	6.52
**Resibufogenin**	0	0	0	0	0	0.55	0	1	1	5.98
**Desacetylcinobufagin**	0	0	0	0	0	0.55	0	1	1	6.25
**Telocinobufagin**	0	0	0	0	0	0.55	0	0	1	5.66

Supplementary Table S1 In silico ADME analysis of the bufadienolides investigated in this study using SwissADME.
